# Supplementary figures and images for: Senescence of endplate osteoclasts induces sensory innervation and spinal pain
Source: bioRxiv. 2024 Jun 25:2023.10.26.564218. Originally published 2023 Oct 26. Preprint. [Version 4] doi: 10.1101/2023.10.26.564218 (PMC10634856; doi:10.1101/2023.10.26.564218)

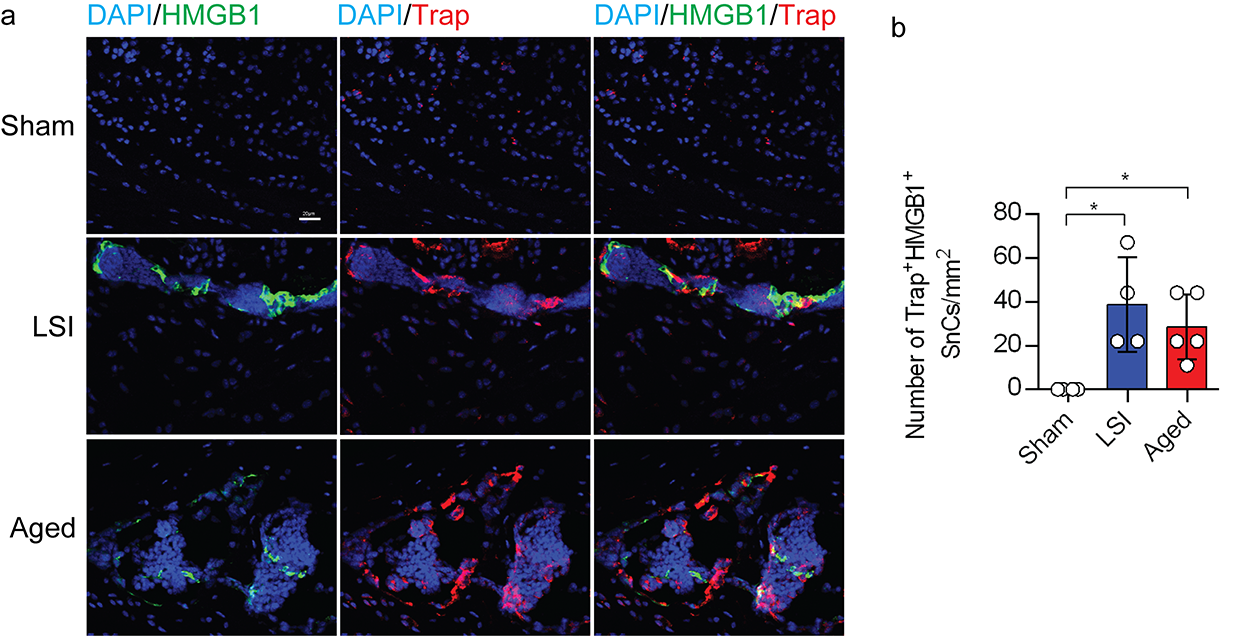

Supplement: Supplement 1 — Figure 1-figure supplement 1. (a) Representative images of immunofluorescent analysis of HMGB1, a senescent marker (green), Trap, an osteoclast marker (red) and nuclei (DAPI; blue) of adult sham, LSI and aged mice. (b) Quantitative analysis of the number of Trap+HMGB1+ SnOCs per mm2. n ≥ 4 per group. Statistical significance was determined by one-way ANOVA, and all data are shown as means ± standard deviations. [file media-1.tif]

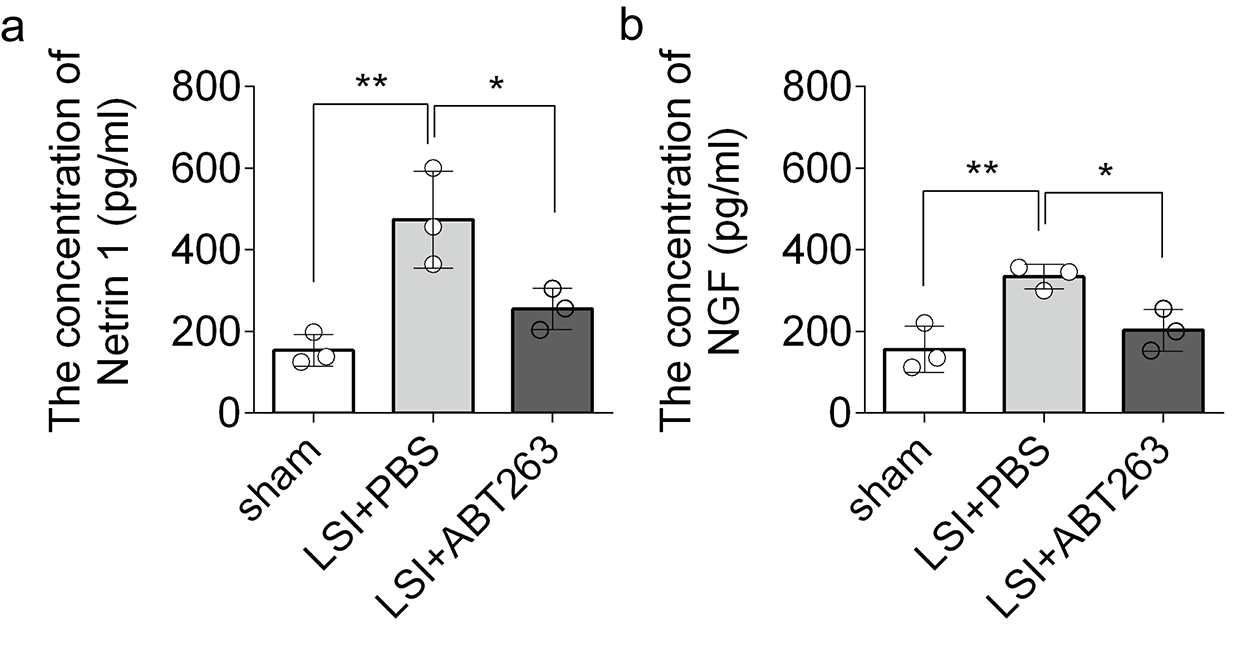

Supplement: Supplement 2 — Figure 6-figure supplement 1. (a) ELISA analysis showing the concentration of Netrin1 in L3–5 endplates of adult sham, LSI+PBS and LSI+ABT263 mice. (b) ELISA analysis showing the concentration of NGF in L3–5 endplates of adult sham, LSI+PBS and LSI+ABT263 mice. n = 3 per group. Statistical significance was determined by one-way ANOVA, and all data are shown as means ± standard deviations. [file media-2.tif]
