# Supplementary material for: Senescence of endplate osteoclasts induces sensory innervation and spinal pain
Source: bioRxiv. 2024 Mar 1:2023.10.26.564218. Originally published 2023 Oct 26. Preprint. [Version 2] doi: 10.1101/2023.10.26.564218 (PMC10634856; doi:10.1101/2023.10.26.564218)
Supplement: Supplementary file 1 [file media-1.pdf]

# Senescence of endplate osteoclasts induces sensory innervation and spinal pain

## Supplementary Figures and Legends

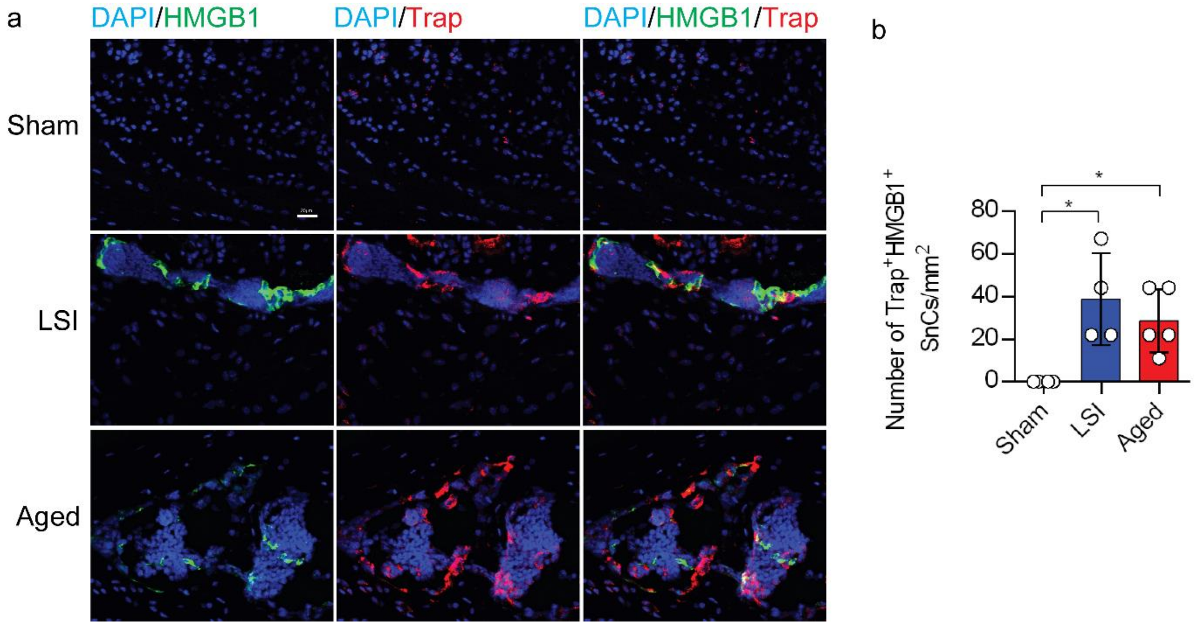


**Supplementary Fig. 1.** (**a**) Representative images of immunofluorescent analysis of HMGB1, a senescent marker (green), Trap, an osteoclast marker (red) and nuclei (DAPI; blue) of adult sham, LSI and aged mice. (**b**) Quantitative analysis of the number of $Trap^{+}HMGB1^{+}$ SnOCs per $mm^2$. $n \geq 4$ per group. Statistical significance was determined by one-way ANOVA, and all data are shown as means ± standard deviations.

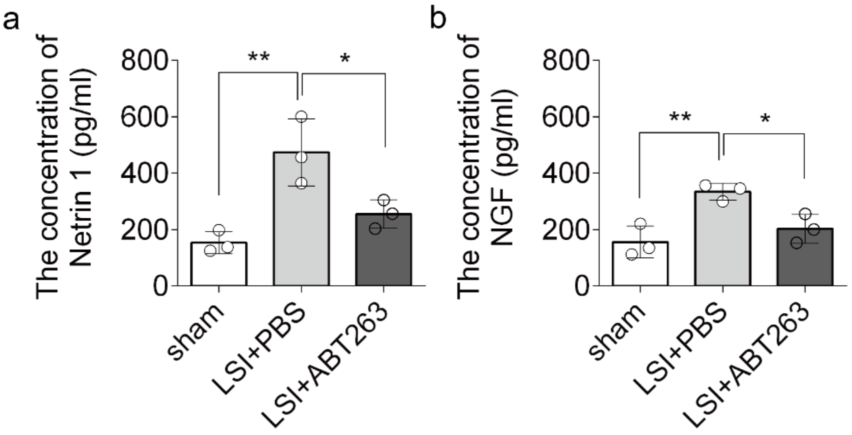


**Supplementary Fig. 2.** (**a**) ELISA analysis showing the concentration of Netrin1 in L3-5 endplates of adult sham, LSI+PBS and LSI+ABT263 mice. (**b**) ELISA analysis showing the concentration of NGF in L3-5 endplates of adult sham, LSI+PBS and LSI+ABT263 mice. *n* = 3 per group. Statistical significance was determined by one-way ANOVA, and all data are shown as means ± standard deviations.
